# Jugular Foramen Syndrome Caused by Varicella Zoster Virus Infection

**DOI:** 10.1177/19418744221116717

**Published:** 2022-08-29

**Authors:** Kwame O. Adjepong, Sara C. LaHue, Deborah Ha, Brandon B. Holmes

**Affiliations:** 1 Department of Neurology, School of Medicine, University of California, San Francisco, CA, USA; 2 Department of Neurology, Weill Institute for Neurosciences, 189226 University of California, San Francisco, CA, USA; 3 Rehabilitative Services, 8785 University of California San Francisco, San Francisco, CA, USA

**Keywords:** cranial neuropathy, dysphonia, jugular foramen, varicella zoster virus, vernet syndrome

## Abstract

Jugular foramen syndrome (JFS) is a lower cranial neuropathy syndrome characterized by dysphonia and dysphagia. The syndrome is caused by dysfunction of the glossopharyngeal, vagus, and spinal accessory nerves at the level of the pars nervosa and pars vascularis within the jugular foramen. There are numerous etiologies for JFS, including malignancy, trauma, vascular, and infection. Here, we present the case of a healthy adult man who developed JFS secondary to an atypical presentation of Varicella Zoster meningitis, and was promptly diagnosed and treated with rapid symptom resolution. We diagnosed the patient using specialized skull-based imaging which detailed the jugular foramen, as well as CSF analysis. This case highlights the clinical value of detailed structural evaluation, consideration for infection in the absence of systemic symptoms, and favorable outcomes following early identification and treatment.

## Introduction

Jugular foramen syndrome (JFS) is a rare lower cranial polyneuropathy characterized by dysphonia and dysphagia. Maurice Vernet, MD described this clinical pattern in 1918 as one of several lower cranial nerve syndromes.^1^ Anatomically, the jugular foramen is formed by the petrous portion of the temporal bone anteriorly, the occipital bone posteriorly, and is divided by a fibrous septum. The anteromedial portion (pars nervosa) contains the glossopharyngeal nerve, while the posterolateral portion (pars vascularis) contains the vagus and spinal accessory nerves, as well as the internal jugular vein.^2^ The most common etiologies of JFS include malignancy, trauma, vascular, inflammation, and infection, although their exact prevalence is unknown due to the rarity of the condition.^3^ Varicella zoster is one recognized etiology for JFS. The infection is usually accompanied by symptoms of meningismus or zoster, as seen in VZV meningitis.^4,5^

In this report, we chronicle the case of a healthy 51-year-old patient who presents with isolated and progressive dysphonia and dysphagia. His neurological examination revealed multiple cranial neuropathies. Specialized cranial nerve and skull-based imaging as well as CSF evaluation revealed acute and localized varicella zoster meningitis in the absence of systemic or typical meningitis features.

## Case Description

A 51-year-old healthy man developed progressive dysphagia and dysphonia. On the first day of symptoms, he endorsed throat tightening with liquids and coughing when swallowing solid food. He presented to a local emergency department and was treated for esophagitis with empiric famotidine. Over the next 4 days, his dysphagia progressed, limiting his oral intake to gelatin and soup. He then developed hoarseness and hiccups. An otolaryngologist performed a laryngoscopy, revealing left vocal cord paresis. He returned to the emergency department where a CT neck with contrast and MRI Brain with contrast were reported as unremarkable. A barium swallow evaluation revealed pooling of oral contrast in the hypopharynx and pyriform sinus, prompting nasogastric tube placement for nutrition. At this time, 7 days from symptom onset, he was transferred to our facility for further evaluation and management.

**Figure 1.**
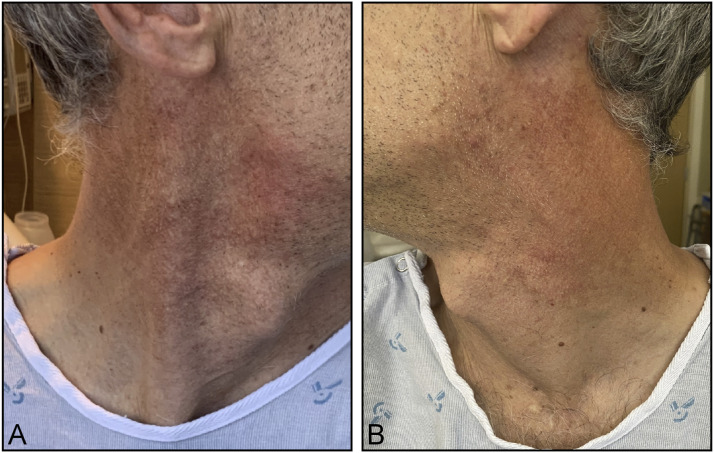
Sternocleidomastoid activation on day 7 from symptom onset. (A) Normal activation of the right sternocleidomastoid muscle. (B) Absent activation of the left sternocleidomastoid muscle.

**Figure 2.**
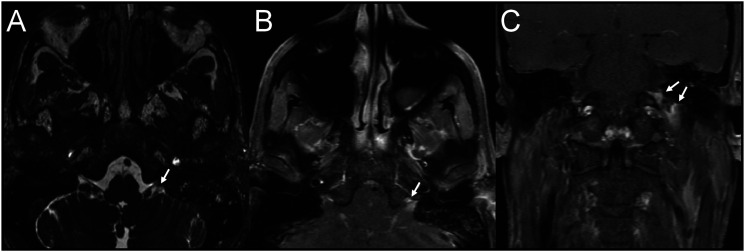
MRI with contrast and Fast Imaging Employing Steady- State Acquisition (FIESTA) sequencing. (A) Axial FIESTA sequence revealing obscuration of the jugular foramen (white arrow). Axial (B) and Coronal (C) T1 post-contrast images revealing enhancement of the left jugular foramen (white arrows).

Upon arrival, his general examination was notable for an eight-pound weight loss since his initial emergency department visit. His neurological examination revealed left palate paralysis, absent left sternocleidomastoid contraction (Figure 1), impaired gustation in the posterior third of the tongue, left scapular winging, and dysphonia. A contrast-enhanced MRI and MRA of the head and neck with skull-based sequencing revealed asymmetric enhancement and obscuration of the jugular foramen, glossopharyngeal and vagus nerves, and intact vasculature (Figure 2). A dedicated CT temporal bone study with contrast revealed enhancement of the left glossopharyngeal recess without osseus lesions. A repeat CT neck revealed asymmetric decrease of the left sternocleidomastoid with medialization of the left vocal fold. Review of his prior imaging revealed a limited view of the jugular foramen. CSF analysis demonstrated colorless fluid with an opening pressure of 13 cm H_2_0, glucose concentration of 45 mg/dL (serum glucose, 104 mg/dL), protein concentration of 54 mg/dL (normal range, 15-45 mg/dL), 69 white blood cells (90% lymphocytes, 7% monocytes, 3% atypical lymphocytes), and 0 red blood cells. VZV DNA PCR returned positive; VZV immunoglobulin M returned positive at an immunological status ratio of 1.22 (reference >1.1 positive) and VZV immunoglobulin G returned positive at 238.3 (reference >165 positive).

Given the lymphocytic pleocytosis and evidence of varicella infection, we diagnosed this patient with acute VZV meningitis and initiated treatment with intravenous acyclovir 10 mg/kg every 8 hours for 14 days. Speech therapy was consulted for evaluation and management of the severe dysphagia. A repeat barium swallow study demonstrated left sided residuals within the pyriform sinus; these residuals were successfully cleared with an accompanying left head tilt during deglutition. The nasogastric tube was removed and the patient was discharged 4 days after initial transfer. At the time of discharge, the dysphonia, hiccups and dysphagia had significantly improved; at the two-week clinic follow-up, the patient’s symptoms completely resolved and his exam returned to baseline.

## Discussion

We present a case of a healthy 51-year-old immunocompetent man with progressive dysphagia and dysphonia caused by focal VZV infection within the jugular foramen. His neurologic examination revealed cranial palsies of the glossopharyngeal, vagus, and spinal accessory nerves suggesting involvement of both the pars nervosa and pars vascularis. Radiographically, skull-based MR imaging disclosed enhancement in the left jugular foramen thereby linking the clinical and anatomic features of this presentation. A diagnosis of VZV meningitis was established through CSF analysis which revealed a lymphocytic pleocytosis and VZV-positive PCR and antibodies. The patient was promptly treated with intravenous acyclovir with rapid and complete symptom resolution within 2 weeks.

Notably, this case illustrates an atypical presentation of varicella zoster meningitis characterized by an isolated jugular foramen syndrome without associated meningismus or zoster. Other case reports identify varicella zoster causing jugular foramen syndrome with associated vesicular rash^6^ or atypical anatomy of the jugular foramen.^7^ Our report combines exam findings with comprehensive imaging and CSF analysis to identify this syndrome in an otherwise anatomically typical and healthy individual. We attribute the lack of systemic signs and symptoms to the highly focal nature of this patient’s VZV infection, localizing to the jugular foramen without extension to the surrounding cranial nerves. As such, an infectious etiology of jugular foramen syndrome should not be prematurely excluded prior to full CSF analysis, even in the absence of other manifestations of infection.

This case required advanced imaging techniques to visualize the complex anatomy of the jugular foramen and associated structures. We used FIESTA (Fast Imaging Employing Steady State Acquisition)––a steady-state gradient echo sequence. This radiographic sequence increases spatial resolution between cranial nerve and cerebrospinal fluid,^8^ with an acquisition time of less than 5 minutes. Linn et al, demonstrated that FIESTA successfully depicts the anatomy of the jugular foramen and differentiates CN IX from the CN X/XI complex compared to standard MRI techniques.^9^ Contrast-enhanced FIESTA sequences have been identified as reproducible and reliable in demonstrating the distinct anatomic courses of structures through the jugular foramen. When paired with a localizing neurologic exam, this sequence can serve as a valuable diagnostic tool in identifying etiologies such has malignancy, inflammation, and infection that may otherwise be missed.^10^ We find this benefit outweighs the disadvantage of the added MRI acquisition time. Of note, we reviewed the patient’s initial brain MRI with contrast, and found this did not provide diagnostic views of the jugular foramen.

Following initial infection, varicella zoster virus becomes latent in ganglionic neurons throughout the neuraxis. As a result, there are myriad neurologic phenotypes of VZV reactivation––zoster, post-herpetic neuralgia, retinal necrosis, meningitis, meningoencephalitis, meningoradiculitis, cerebellitis, myelopathy, and vasculopathy.^11^ Zoster does not always precede neurologic complications and therefore diagnosis may require CSF evaluation.^4^ VZV PCR can be a useful confirmatory test for CNS infection, however its sensitivity is only 30%.^12^ The addition of VZV antibody testing, including IgM and IgG, increases the diagnostic sensitivity to over 90% and can suggest acute infection.^12,13^ We conclude that in acute presentations, diagnostic evaluation of VZV in the CNS should always include testing by PCR as well as immunoglobulin M and immunoglobulin G.^11,12^

Varicella zoster is a treatable cause of jugular foramen syndrome with variable presentations that require thorough diagnostic evaluation including dedicated skull-based imaging and CSF analysis with DNA and immunoglobulin testing. We provide a diagnostic framework for this rare condition. Prompt diagnosis and initiation of therapy may minimize the morbidity associated with JFS.
